# Discrepancy Between ASTER- and MODIS- Derived Land Surface Temperatures: Terrain Effects

**DOI:** 10.3390/s90201054

**Published:** 2009-02-17

**Authors:** Yuanbo Liu, Yousuke Noumi, Yasushi Yamaguchi

**Affiliations:** 1 Nanjing Institute of Geography and Limnology, Chinese Academy of Sciences, 73 East Beijing Road, Nanjing 210008, China; 2 Faculty of Informatics, Okayama University of Science, Okayama 700-0005, Japan; E-Mail: y_noumi@big.ous.ac.jp; 3 Graduate School of Environmental Studies, Nagoya University, Furo-cho, Chikusa-ku, Nagoya 464-8601, Japan; E-Mail: yasushi@nagoya-u.jp

**Keywords:** Land surface temperature, terrain effects, surface emissivity, ASTER, MODIS

## Abstract

The MODerate resolution Imaging Spectroradiometer (MODIS) and the Advanced Spaceborne Thermal Emission Reflection Radiometer (ASTER) are onboard the same satellite platform NASA TERRA. Both MODIS and ASTER offer routine retrieval of land surface temperatures (LSTs), and the ASTER- and MODIS-retrieved LST products have been used worldwide. Because a large fraction of the earth surface consists of mountainous areas, variations in elevation, terrain slope and aspect angles can cause biases in the retrieved LSTs. However, terrain-induced effects are generally neglected in most satellite retrievals, which may generate discrepancy between ASTER and MODIS LSTs. In this paper, we reported the terrain effects on the LST discrepancy with a case examination over a relief area at the Loess Plateau of China. Results showed that the terrain-induced effects were not major, but nevertheless important for the total LST discrepancy. A large local slope did not necessarily lead to a large LST discrepancy. The angle of emitted radiance was more important than the angle of local slope in generating the LST discrepancy. Specifically, the conventional terrain correction may be unsuitable for densely vegetated areas. The distribution of ASTER-to-MODIS emissivity suggested that the terrain correction was included in the generalized split window (GSW) based approach used to rectify MODIS LSTs. Further study should include the classification-induced uncertainty in emissivity for reliable use of satellite-retrieved LSTs over relief areas.

## 1. Introduction

The Advanced Spaceborne Thermal Emission Reflection Radiometer (ASTER) and the MODerate-resolution Imaging Spectroradiometer (MODIS) are two of the five scientific instruments onboard the satellite platform, Terra, part of NASA's Earth Observation System (EOS). It was launched on December 18, 1999 and began collecting data on February 24, 2000 [1]. MODIS provides multi-spectral data at 250-m, 500-m, and 1-km resolutions with almost daily coverage of the Earth, which is invaluable for both local and global change research [2]. ASTER collects multi-spectral data at high spatial resolution from 15-m to 90-m with a 16-day recurrent cycle for geological and environmental applications [3]. Being onboard the same satellite platform, ASTER and MODIS are complementary in spatial and temporal resolutions. This feature offers a unique opportunity for comparative study of retrieval algorithms and investigation of scale-relevant issues.

Either ASTER or MODIS provides data are used for routine retrieval of land surface temperatures (LSTs). ASTER LST has a spatial resolution of 90-m with coverage of 60-km by 60-km. The temperature and emissivity separation (TES) algorithm allows accurate retrieval of LST products using the ASTER multi-spectral thermal infrared (TIR) bands [4]. MODIS LST is retrieved from the generalized split window (GSW) or the day/night LST algorithms, which are view-angle dependent [5-7]. The GSW algorithm retrieves 1-km LST, and the day/night algorithm retrieves 5-km LST products. Both ASTER and MODIS LST products have been widely used in meteorological, hydrological, and ecological studies [8-10].

Although simultaneous observation eliminates the differences in ASTER and MODIS LST data due to time differences, recent studies have reported a discrepancy in ASTER and MODIS LSTs by approximately 3 K over semi-arid areas [7,11,12]. The ASTER-to-MODIS LST discrepancy can be ascribed to the differences in spatial resolution and the retrieval algorithms used [11,13]. Indeed, satellite-retrieved LST is an ensemble quantity representing the integrated effects of temperature variations within a pixel [14-15]. In regard to resolution difference, Jacob *et al.* compared ASTER and MODIS LSTs over a semi-arid and a Savannah area, and found no significant ASTER-to-MODIS LST differences caused by spatial heterogeneity [13]. Using a scaling approach explicitly accounting for resolution difference, Liu *et al.* showed that spatial heterogeneity made the effects negligible [11]. With respect to the retrieval algorithm, Wan *et al.* developed a correction approach to rectify the underestimated MODIS 1-km LST product [7]. Recently, Liu *et al.* used the Wan *et al.*'s approach to reduce the LST discrepancy between ASTER-to-MODIS [11]. They further refined the Wan *et al.*'s approach using the Planck function and ASTER emissivity data. In addition, Liu *et al.* proposed another correction approach based on the principle of the GSW algorithm. So far, all the correction techniques showed the improved agreement between ASTER and MODIS LST products [12].

Both ASTER and MODIS LST products have been applied worldwide. It is well-known that a large fraction of the earth surface consists of mountainous areas. Variations in elevation, terrain slope and aspect angles can interact with satellite viewing geometry to cause biases in retrieved LSTs [16]. Therefore, the impact of topography on remote sensing data should be examined prior to any remote sensing applications [17]. In regard to the ASTER and MODIS LST products, only the elevation effect was corrected in the routine retrieval [4,6]. Neither ASTER nor MODIS LST was corrected for terrain angular and adjacency effects. This may generate the biases to the ground truth and, subsequently, the discrepancy between ASTER and MODIS LST values. By accounting for heterogeneity and terrain effects on ASTER LST using a physics-based approach, Liu *et al.* found that terrain effects contributed approximate 0.7 K to the ASTER-to-MODIS discrepancy in a rugged area on the Loess Plateau of China [11]. However, they did not fully address the discrepancy relevant to terrain effects. A more detailed explanation is necessary to enhance our understanding on the role of terrain in LST products. Clarification of the uncertainties relevant to terrain features would enhance our confidence in use of LST products over rugged areas.

In this study, we use the data sets same as Liu *et al.* [12] to analyze terrain effects on LST, and to provide a more detailed documentation to the terrain-induced discrepancy between ASTER and MODIS LSTs. Section 2 introduces methods for dealing with the LST discrepancy between ASTER-to-MODIS. Section 3 describes study materials and data processing. Section 4 analyzes results and discusses the terrain-induced effects.

## 2. Methods

In the study, we adopt three approaches developed in [11,12]. One approach is to correct the terrain effects remained in satellite-derived LST products. The second one is an upscaling approach. It deals with topographic heterogeneity, in which includes the terrain angular and adjacency effects. Upscaling of ASTER LST into the nominal resolution of MODIS allows ASTER and MODIS LST to be comparable at the same spatial resolution. The third one is a GSW algorithm based correction approach. It is used to rectify the MODIS 1-km LST products, as being complementary in evaluating the terrain-induced effects on MODIS LST.

### 2.1. Terrain correction

Both ASTER and MODIS LST products were not corrected for terrain angular and adjacency effects. The terrain-induced angular effect can be corrected based on the cosine method [18] as follows [19]:

(1) $$T={\left(\frac{T\prime^{4}}{\cos\gamma }\right)}^{1/4}$$

where *T* is the corrected LST and *T*′ the satellite-derived LST. *γ* is the angle between the satellite-view path and the normal to the terrain element. To a thermal band, the angle of emitted radiance can be geometrically determined from the following equation:

(2) $$\cos\gamma =\cos\alpha \cos\beta +\sin\alpha \sin\beta \cos(\phi_{s}-\phi )$$

where *α* is the local slope angle, *β* the satellite zenith angle, *φ_s_* the satellite azimuth angle, and *φ* the aspect angle of the terrain element.

In addition to the emittance by the terrain element, the terrain irradiance is also contributed from the adjacent terrain [20-22]. The adjacency effect on the terrain irradiance is accounted for by the terrain-view factor with a trigonometric function as:

(3) $$M_{a}=\pi \bar{L}\frac{1-\cos\alpha }{2},$$

where *M_a_* is the irradiance from the adjacent terrain and *L(x00304)* is the local average radiance emitted from the surrounding terrain [23]. The effect needs to be included in terrain correction for satellite data with a fine spatial resolution. In the case of MODIS data with a spatial resolution of 1-km, it is negligible [24].

### 2.2. Upscaling of satellite-retrieved LST

ASTER LST has a spatial resolution of 90-m, different from MODIS LST, which are directly incomparable with respect to spatial resolution. It is necessary to upscale ASTER LST before a comparison of ASTER and MODIS data. ASTER LST can be upscaled with a scaling function from a fine resolution into a coarse one. In the case of topographic heterogeneity, the angular and adjacency effects can be accounted for in the follow scaling function [11]:

(4) $$T={\left(\frac{2\sum {ɛ}_{i}T_{i}^{4}\sec\alpha_{i}-\pi \bar{L}\sum (\sec\alpha_{i}-1)\cos\gamma_{i}}{2ɛ\cos\gamma \sum \sec\alpha_{i}}\right)}^{1/4}$$

where the subscript *i* denotes the pixel *i* at a sub-pixel or fine scale. The upscaled ASTER LST may serve as a basis for investigating the uncertainty embedded in MODIS 1-km LST.

### 2.3. GSW algorithm based correction approach

The MODIS 1-km LST products have been found to be underestimated with the GSW algorithm [25] over semiarid areas [7, 11, 12]. To reduce the underestimation, Liu *et al.* proposed a correction approach as follows [19]:

(5) $$T_{s}^{\prime }=T_{s}+\underset{a}{\underset{︸}{\left(A_{2}\frac{T_{31}+T_{32}}{2}+B_{2}\frac{T_{31}-T_{32}}{2}\right)}}(\frac{1}{ɛ\prime }-\frac{1}{ɛ})+\underset{b}{\underset{︸}{\left(A_{3}\frac{T_{31}+T_{32}}{2}+B_{3}\frac{T_{31}-T_{32}}{2}\right)}}\Delta ɛ(\frac{1}{ɛ\prime^{2}}-\frac{1}{{ɛ}^{2}})$$

where *ε* = 0.5(*ε*_31_ + *ε*_32_) and Δ*ε* = *ε*_31_ − *ε*_32_. *T_s_* is the retrieved LST (K). *T*_31_ and *T*_32_ are the brightness temperature (K), and *ε*_31_ and *ε*_32_ are the emissivity used for MODIS TIR bands 31 and 32, respectively. *A*_2_, *A*_3_, *B*_2_, and *B*_3_ are the coefficients in the look-up table defined by Wan and Dozier [25]. $ɛ\prime =0.5({ɛ}_{31}^{\prime }+{ɛ}_{32}^{\prime })$. $T_{s}^{\prime }$ is the rectified LST (K). ${ɛ}_{31}^{\prime }$ and ${ɛ}_{32}^{\prime }$ are the “accurate” emissivities. The components ***a*** and ***b*** can be estimated from multiple regression approach [19].

With the three approaches, we can reduce the discrepancy between ASTER and MODIS LST. Further incorporated with angular and surface features, we may make more insightful analysis into terrain effects on MODIS LST.

## 3. Study materials and data processing

### 3.1. Study area and materials

**Figure 1.**
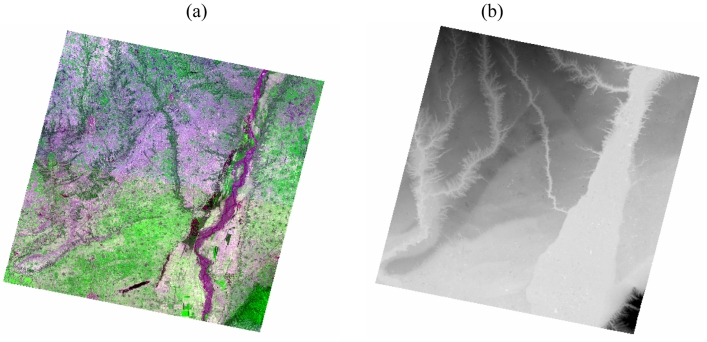
(a) The false color image and (b) DEM of ASTER (RGB: band 132) for the study area, dated on 8 June 2004.

The study area is located on the Loess Plateau of China. Figure 1 shows the land cover features and topography of the area, produced from ASTER data collected on 8 June 2004. The geographic extent was defined by the acquired ASTER image with four corners: (34.44°N, 109.47°E), (34.21°N, 110.27°E), (33.65°N, 110.14°E), and (33.78°N, 109.35°E). Agricultural field, grassland, bare soil surface, forestland, and inland water surface dominant the area [Figure 1(a)]. It has highly variable topographical features suffering from serious soil erosion in the plateau. The elevation of the area ranges from 327 to 1,430 m, with an average of 518.6 m [Figure 1(b)]. The mean slope is 2.8 degrees with a standard deviation (S.D.) of 4.5 degrees, and the maximum slope is 43.6 degrees.

**Figure 2.**
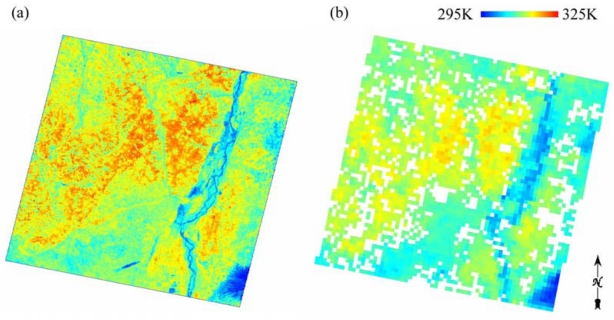
(a) The ASTER LST image and (b) MODIS image for the study area, dated on 8 June 2004.

We used ASTER surface emissivity (AST_05) and surface kinetic temperature (AST_08) products, dated on 8 June 2004. Liu *et al.* [11, 12] detailed these products. Figure 2(a) shows the acquired ASTER LST image. In addition, we used 3D-Ortho product (AST3A01) served as the digital elevation model (DEM) in this study. The 1-km DEM data were generated from the 90-m DEM data [11].

We acquired the same date MODIS products including MOD02_QKM, MOD02_HKM, MOD02_1KM, MOD03_L1A, and MOD11_L2 data. MOD03_L1A geolocation product provided satellite zenith and azimuth angles for each MODIS 1-km pixel. MOD11_L2 data contains LST and band-averaged emissivity in band-31 (10.780–11.280 μm) and band-32 (11.770–12.270 μm) generated using the GSW algorithm. Figure 2(b) shows the segmented MODIS LST image as the ASTER coverage. The scattered white pixels were missing values in the MODIS LST products. Other products were detailed in [11,12].

### 3.2. Data Processing

MODIS has the geolocation with an accuracy of 18±38 m in-track and 4±40 m cross-track [26]. ASTER has the geolocation with an accuracy of 50±15 m in- and cross-track [27]. Liu *et al.* [11,12] detailed image co-registration and segmentation.

ASTER narrowband emissivity was produced from bands 13 and 14 emissivity data. It was used as “accurate emissivity” in LST correction [13]. The narrow-band emissivity data were upscaled from 90-m to 1-km within the footprint of the corresponding nominal MODIS pixel [10]. The broadband emissivity was generated from all the emissivity in the five bands in the AST_05 product. It was used to upscale ASTER LST from 90-m to 1-km using equation (4). The upscaled LST was used as a reference for exploring the uncertainty embedded in MODIS 1-km LST. The 1-km LST data from MODIS product was rectified using [equation (5)]. The rectified MODIS LST was further corrected for terrain angular effect using equation (1).

Essentially, MODIS LST is determined from surface emissivity in the GSW algorithm. To evaluate the uncertainty in emissivity, we compared the ASTER and the MODIS emissivity at the corresponding wavelength. To investigate the uncertainties related to surface feature, we generated LST difference images from ASTER LST to original MODIS LST, and to that rectified with or without terrain correction. To find the terrain-induced uncertainty in MODIS LST, we analyzed the relationships of MODIS-to-ASTER LST and emissivity with angular matrix (angle of local slope and angle of emitted radiance)

## 4. Results and Discussion

We first report the MODIS-to-ASTER LST discrepancy, which served as a basis for subsequent analysis. With concern on the relationships between MODIS LST and terrain feature, we then analyze the MODIS-to-ASTER discrepancy relevant to angle of local slope and angle of emitted radiance, in addition to spatial distribution of the discrepancy. At last, we address the uncertainty in MODIS emissivity, which directly determines the accuracy of MODIS LST.

### 4.1. The discrepancy between the original MODIS and the upscaled ASTER LST

**Figure 3.**
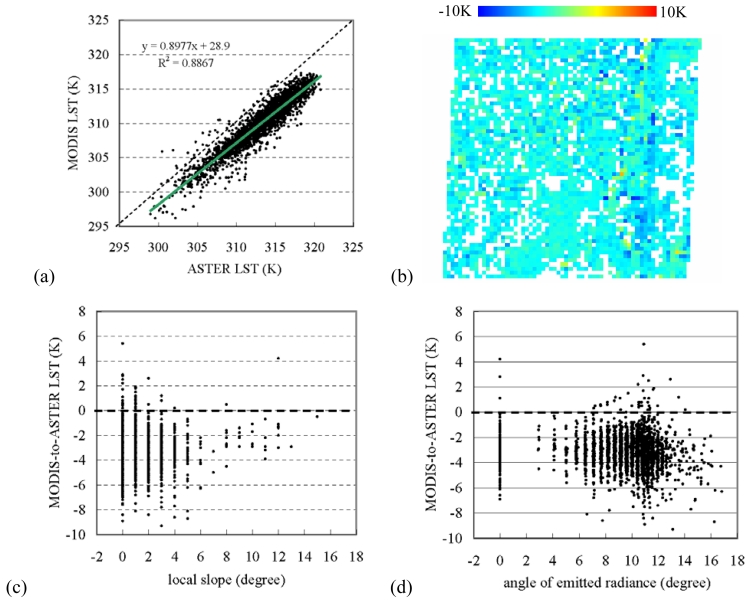
(a) ASTER LST versus original MODIS LST, (b) spatial distribution of the ASTER-to-MODIS LST discrepancy, (c) local slope versus ASTER-to-MODIS LST, and (d) angle of emitted radiance versus ASTER-to-MODIS LST, for the study area.

The original 1-km MODIS LST was general lower than the ASTER LST. It had a discrepancy to the upscaled ASTER LST with -2.7±1.28K and a root-mean-square-error (RMSE) of 3.02K [Figure 3(a)]. The major large differences were located at the eastern part of the area along the edge of the valley [Figure 3(b)]. However, this does not mean that a large local slope would necessarily lead to a large LST discrepancy. Indeed, the ASTER-to-MODIS LST discrepancy had a larger range at a small slope than at a large slope [Figure 3(c)]. There was no significant trend in the LST discrepancy varying with the local slope. In contrast, the ASTER-to-MODIS LST difference was general lower than zero, and it had a tendency to change with the angle of emitted radiance (*p*>0.005). The largest difference was at an angle of around 11 degree [Figure 3(d)]. This demonstrated that the angle of emitted radiance was more important than the slope angle in affecting LST discrepancy.

### 4.2. The discrepancy between the rectified MODIS and the upscaled ASTER LST

**Figure 4.**
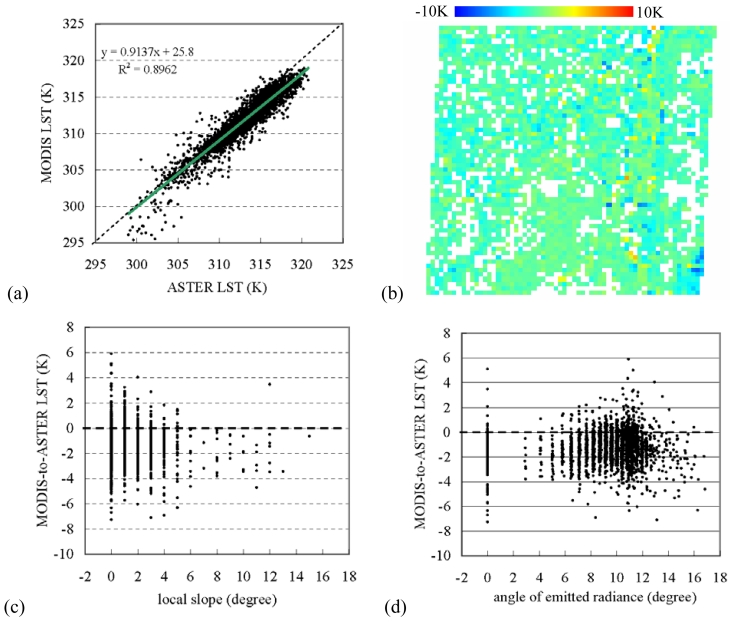
(a) ASTER LST versus the rectified MODIS LST, (b) spatial distribution of ASTER-to-MODIS LST discrepancy, (c) local slope versus ASTER-to-rectified MODIS LST, and (d) angle of emitted radiance versus ASTER-to-rectified MODIS LST.

The original MODIS LST increased generally after rectification with the GSW based approach. Yet, the discrepancy between the rectified LST and the ASTER is as large as 1.2±1.21 K. Even though the S.D. was lower than that of the ASTER-to-original MODIS LST, there existed the relative large differences at the range of 298-305K [Figure 4(a)]. These pixels were distributed mainly at the eastern part of the area [dark blue pixels in Figure 4(b)]. In comparison with Figure 1(a), these pixels are densely vegetated areas with high emissivity. Anyway, the LST discrepancy was close to zero mean from a low to a high slope [Figure 4(c)]. Comparing Figure 4(d) with Figure 3(d), we can see that the distribution of LST difference also changed with the angle of emitted radiance. This suggested that, even without explicit inclusion of terrain factors in the rectification, the original MODIS LST was rectified with the ASTER emissivity in which included already terrain effect.

### 4.3. The discrepancy between the rectified MODIS LST with terrain correction and the upscaled ASTER LST

**Figure 5.**
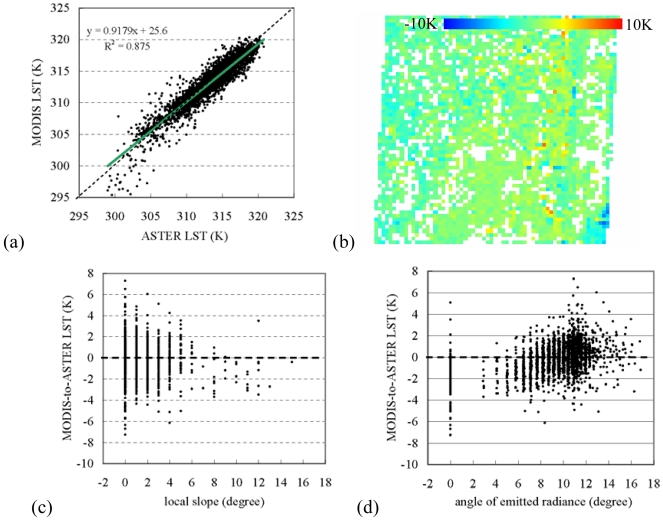
(a) ASTER LST versus rectified MODIS LST with terrain correction, (b) spatial distribution of ASTER-to-MODIS LST discrepancy, (c) local slope versus ASTER-to-MODIS LST, and (d) angle of emitted radiance versus ASTER-to-MODIS LST.

Further corrected for terrain effect, the discrepancy was reduced to 0.1±1.33 K on average between ASTER and MODIS LST. Likely, there existed some pixels with a relative large difference at the range of 298-305K [Figure 5(a)]. The pixels were mainly vegetated areas with a high emissivity close to unity. It suggested that the conventional terrain correction might be not sensitive to and unsuitable for vegetated areas. To date, few terrain correction approaches incorporated the Bidirectional Reflectance Distribution Function (BRDF) effects, which is more significant to vegetation [28]. A more specific correction needs to be developed for densely vegetated rugged area. With respect to angular effect, the mean of LST difference was generally close to zero within the range of local slope angle. In contract, the LST discrepancy reduced more with a larger angle of emitted radiance [Figure 5(d)], because the terrain correction took effective directly with the angle of emitted radiance rather than the angle of local slope [equation(1)]. Again, the LST discrepancy showed a tendency to change with the angle of emitted radiance, as a result of the comprised angular and emissivity effects (Section 4.4). Further compared with the original MODIS-to- ASTER LST (Section 4.1), the direct terrain-induced effect contributed approximately 30% to the total LST discrepancy. Overall, it made not the major but the important influence on the MODIS 1-km LST.

### 4.4. Terrain effect relevant to emissivity

**Figure 6.**
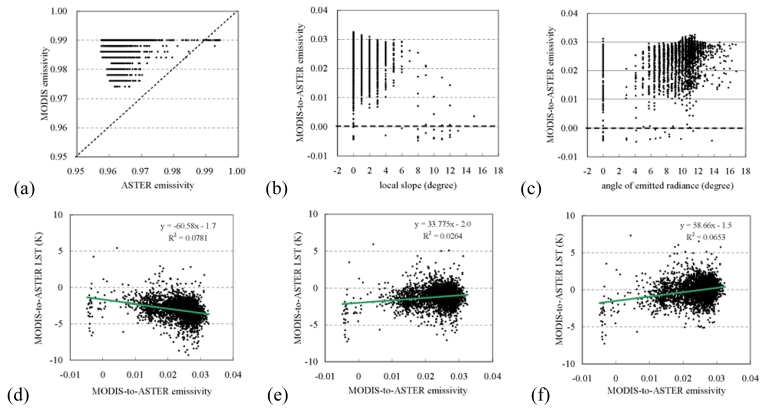
(a) ASTER narrowband emissivity versus MODIS emissivity (band 31), (b) local slope versus ASTER-to-MODIS emissivity, (c) angle of emitted radiance versus ASTER-to-MODIS emissivity, (d) MODIS-to-ASTER emissivity versus LST without any correction, (e) MODIS-to-ASTER emissivity versus LST rectified with GSW based approach, and (f) MODIS-to-ASTER emissivity versus the rectified LST with terrain correction.

MODIS emissivity is the key to retrieval of the MODIS 1-km LST. The uncertainty in emissivity directly affects the accuracy of LST. In the present examination, MODIS emissivity in band 31 had a large discrepancy to the corresponding ASTER emissivity and they showed no obvious correlation [Figure 6(a)]. As a result, the overestimated MODIS emissivity led to the underestimated LST.

With respect to terrain features, the ASTER-to-MODIS emissivity difference showed no obvious relation with local slope angle [Figure 6(b)]. In contrast, the discrepancy had a stronger trend with the angle of emitted radiance [Figure 6(c)]. This evidence enhanced our inference in Section 4.2 that terrain effect was included in emissivity used for rectifying the original MODIS LST. As a further inference, terrain features might have taken effects on retrieval of emissivity, and subsequent retrieval of LST. As a result, the significance of the relation in Figure 6(d) decreased after the original MODIS LST was rectified (*p*<0.001). Changes in the relationship of emissivity difference versus LST discrepancy supported our inferences on terrain effects [Figures 6(d)-6(f)].

The accuracy of MODIS emissivity relies on classification of land cover [25]. Each class has its designed emissivity value. Misclassification, not necessarily related to topographic feature, unavoidably results in errors in surface emissivity. For reliable use of satellite-retrieved LST, classification-induced uncertainty in emissivity needs to be explored in future study.

## 5. Conclusions

The present study reported the terrain effects on the LST discrepancy between ASTER and MODIS with a case examination over a relief area at the Loess Plateau of China. The direct terrain-induced effect made not the major but was an important influence on the MODIS 1-km LST. A large local slope does not necessary lead to a large LST discrepancy. The angle of emitted radiance was more important than the slope angle in generating the LST discrepancy. Specifically, the conventional terrain correction may be unsuitable for densely vegetated area. The distribution of ASTER-to-MODIS emissivity suggested that the terrain correction was included in the generalized split window (GSW) based approach used to rectify MODIS LST. Further study should include the uncertainty in emissivity, related to classification error, for reliable use of satellite-retrieved LST over relief area.
